# Differential incorporation of one-carbon substrates among microbial populations identified by stable isotope probing from the estuary to South China Sea

**DOI:** 10.1038/s41598-018-33497-6

**Published:** 2018-10-18

**Authors:** Wenchao Deng, Lulu Peng, Nianzhi Jiao, Yao Zhang

**Affiliations:** State Key Laboratory of Marine Environmental Sciences and College of Ocean and Earth Sciences, Xiamen University, Xiamen, 361101 China

## Abstract

Methanol (MOH) and monomethylamine (MMA) are two typical one-carbon (C_1_) compounds found in natural environments. They play an important role in marine and atmospheric chemistry, cloud formation, and global climate. The main biological sink of MOH and MMA is rapid consumption by marine microbes. Here, field-based time-series incubations with supplemental ^13^C-labelled MOH and MMA and isotope ratio analyses were performed. A substantial difference in the MOH and MMA incorporation rates and bacterial taxa were observed between the South China Sea (SCS) and the Pearl River estuary. C_1_ substrates were assimilated more quickly in the estuary than the SCS shelf where MOH and MMA had similar bio-availability. However, microbial responses to MMA may be faster than to MOH in the coastal and basin surface water of the SCS despite similar active bacterial populations. Three ecological types of bacteria, in terms of response to supplemented MOH and MMA, were identified: rapid incorporation (I, dominant C_1_-incorporating group), slow incorporation (II, minor C_1_-incorporating group), and no incorporation (III, C_1_-non-incorporating group). Members of the families *Methylophilaceae* (*β*-Proteobacteria) and *Piscirickettsiaceae* (*γ*-Proteobacteria) belonged to type I and actively incorporated substrates in the estuary and SCS, respectively. Diverse MOH and MMA-incorporating type II bacteria were identified by stable isotope probing in the SCS, and could play a more important role in the transformation of C_1_ compounds in marine environments than hitherto assumed.

## Introduction

One-carbon (C_1_) compounds consist of a single carbon atom (lacking carbon-carbon bonds) such as methane, methanol (MOH), monomethylamine (MMA), halogenated methanes and methylated sulphur^1^. C_1_ compounds exert influence on marine and atmospheric chemistry, cloud formation, and global climate. For example, MOH is an oxygenated volatile organic compound that contributes to rainwater acidity through photochemistry^2,3^; it positively influences the oxidising capacity and ozone-forming potential of the atmosphere, and is thus a climate-relevant gas^4–6^. As one of the most abundant C_1_ compounds on Earth, MOH occurs naturally in the environment since plants release it as they grow and decompose. A recent study found that the main source of MOH in the ocean is phytoplankton, which have a unique ability to produce MOH^7^ in quantities that rival what is produced on land. Experimental models have estimated that there are ~30 and ~100 million tons of MOH released from ocean and terrestrial plants, respectively, into the atmosphere per year^8,9^, and globally ~600 million tons of methane released into the atmosphere per year^10^. MOH is an “alcohol derivative” of methane. However, MOH has a higher solubility than methane and can be easily deposited in the ocean from the atmosphere^8,11^. According to model estimates, the amount of stored MOH in the global ocean is about 228 Tg, 67 times higher than the atmosphere^8^. Thus, the ocean serves as a huge natural reservoir of MOH. Many previous studies showed that concentrations of MOH in seawater ranged from 27 to 429 nM^5^ and decreased with depth^12^. MOH is a ubiquitous source of C_1_ substrate, which aids the widespread distribution of methylotrophic organisms throughout the surface ocean. Despite high volumes, MOH has a short residence time in seawater, suggesting that high production from phytoplankton is offset by rapid consumption. This, combined with its high solubility, suggests that consumption by microorganisms prevents MOH from escaping into the atmosphere in significant quantities.

MMA, another important C_1_ compound in atmospheric and marine environments, is a derivative of ammonia, with one hydrogen atom replaced by a methyl group. MMA is derived from the degradation of compatible solutes, choline, glycine betaine, and trimethylamine that is released by algae, invertebrates, and fish, as they adjust their osmotic pressure^13^. Thus, MMA widely distributed in the ocean^14,15^. The concentration of MMA generally ranges from 0 to 66 nM, with higher values in shoals, off shore, and high productivity areas, and lower values in oligotrophic areas, particularly the deep sea (<10 nM)^16^. As an ammonium analogue, MMA has an inhibiting effect for ammonium oxidation and chemoautotrophic growth of marine nitrifiers^17^. MMA can act as a carbon or nitrogen source for marine microbes, and thus significantly participates in both the carbon and nitrogen cycles^14,15^. Methylotrophs use MMA as a carbon source, while non-methylotrophic microorganisms utilise MMA as a nitrogen source^18^.

As the incorporation of MOH and MMA by microbes (mainly Methanotrophs) is the main biological sink, the microbes involved in this process have been intensively studied^19–21^. Methylotrophs are widespread in a diverse range of habitats, and most of them belong to the classes *Alphaproteobacteria* (*α*-Proteobacteria), *Betaproteobacteria* (*β*-Proteobacteria), and *Gammaproteobacteria* (*γ*-Proteobacteria), the phyla *Actinobacteria*, *Firmicutes*, and *Verrucomicrobia*, as well as a *Candidatus phylum* NC10^22–24^. It has been reported that methanotrophs captured the majority (up to over 95%) of methane produced via methanogenesis or non-biogenically^25,26^, whereas non-methanotrophic methylotrophs constitute important barriers for accumulation of other environmentally important C_1_ compounds, such as MOH, methylated amines, and halogenated methanes^27^. Previous studies on non-methanotrophic methylotrophs have mainly focussed on the enrichment and isolation of methylotrophs^28–30^, but only a small number of microorganisms were available in pure culture. As a cultivation-independent analysis, DNA-based stable isotope probing (DNA-SIP) provides a powerful tool to characterise the metabolic activity of microorganisms under *in-situ* conditions by tracking the stable isotopes from isotopically labelled substrates into DNA^31,32^. Compared with other SIP technologies, such as phospholipid fatty acid (PLFA)-SIP, RNA-SIP and protein-SIP, DNA-SIP can identify a broad spectrum of microorganisms involved in a particular process and with high phylogenetic resolution^33,34^; thus, it can detect organisms that have previously been undetected using other methods^35^. The identity of microorganisms responsible for the transformation of C_1_ compounds, which has not yet been determined, will fill major knowledge gaps regarding the role of these compounds in the marine carbon cycle. However, so far there is very few studies regarding the C_1_ compounds-incorporating microbes in marine systems. Neufeld *et al*.^6,36^ reported MOH and MMA utilization by *Methylophaga* and several clades of unclassified *γ*-Proteobacteria based on DNA-SIP and clone library analysis in the English Channel. Many open questions remain about the MOH and MMA-incorporating microbes, including diversity, incorporation rates, and difference among the typical marine systems.

**Figure 1:**
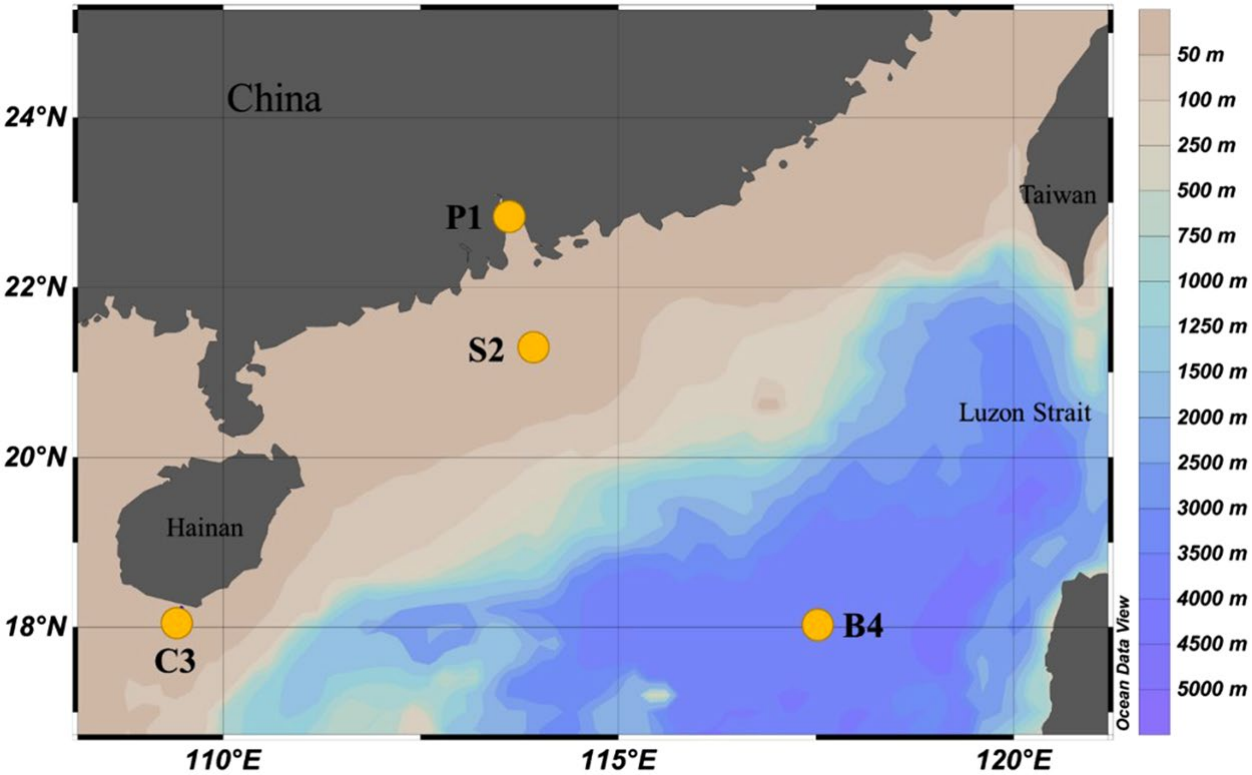
Map of the South China Sea and sampling stations. Isobaths are used as the background and the colour bar indicates water depth. Figure 1 was produced using Ocean Data View v. 4.7.10 (Schlitzer, R., Ocean Data View, odv.awi.de, 2017).

The South China Sea (SCS) is one of the largest marginal seas, with a deep basin in the tropical to subtropical western North Pacific^37^. Here, we carried out field-based time-series incubation experiments with supplemental ^13^C-labelled MOH or MMA in different water masses of the SCS (Table S1), covering the Pearl River estuary (site P1, 113.57°E, 22.90°N), coastal water (site C3, 109.48°E, 18.22°N), continental shelf water (site S2, 113.93°E, 21.34°N), and the oligotrophic central basin (site B4, 117.50°E, 18°N), during two cruises in July–October 2013 (Fig. 1)^38^. Subsequently, ^13^C and ^12^C-DNA separated through ultracentrifugation were analysed by terminal-restriction fragment length polymorphism (T-RFLP) and high-throughput sequencing. In addition, carbon isotopic compositions (*δ*^13^C) of particulate organic carbon (POC) in incubated seawater were measured to estimate ^13^C-labelled substrate assimilation rates. The objectives of this study were to identify the key microbial players involved in the transformation of the two typical C_1_ substrates (MOH and MMA), compare results among the typical water masses of the SCS, and evaluate microbially driven C_1_ cycling in the ocean.

## Results

### ^13^C-labelled substrate assimilation

The assimilation rate of ^13^C-labelled MOH was 165.6 μg L^−1^ d^−1^ after the seawater from 5 m depth of the estuary site P1 was incubated for one day, and decreased to 77.4 μg L^−1^ d^−1^ after four days of incubation. In contrast, samples at the shelf site S2 had a ^13^C-MOH assimilation rate of 14.8 μg L^−1^ d^−1^ (5 m water depth) and 1.4 μg L^−1^ d^−1^ (69 m water depth) after one day of incubation, which increased to 44.0 μg L^−1^ d^−1^ (5 m water depth) and 41.1 μg L^−1^ d^−1^ (69 m water depth) after four days of incubation. Consistent with the MOH assimilation rates of samples at site S2 from one to four days of incubation, the assimilation rate of ^13^C-labelled MMA was distinctly higher after four days than one. The assimilation rates of ^13^C-MMA were higher in samples at 69 m depth than 5 m (Table 1). Overall, there was no significant difference between the assimilation rates of ^13^C-MOH and ^13^C-MMA at site S2. The same trends were observed in the ^13^C percent in total POC (Table 1). It decreased with increasing incubation time of samples at site P1, indicating that some microbially assimilated ^13^C-MOH was degraded to carbon dioxide. The substrate assimilation rates were not measured at sites C3 and B4 as all culture volumes were collected for ultracentrifugation and molecular analysis.

**Table 1:** Microbial assimilation rates of ^13^C-labeled substrates and percent of ^13^C in total particulate organic carbon during incubation experiments. MOH, methanol; MMA, monomethylamine.

Station	Depth (m)	Substrate	Assimilation (^13^C) (μg L^−1^ d^−1^)		^13^C%	
			1 d	4 d	1 d	4 d
P1	5	MOH	165.6	77.4	27.9	17.9
S2	5	MOH	14.8	44.0	10.9	46.5
		MMA	0.4	26.7	0.5	48.2
	69	MOH	1.4	41.1	3.0	51.7
		MMA	11.4	39.8	20.0	58.4

### DNA distribution in CsCl density gradient fractions

**Figure 2:**
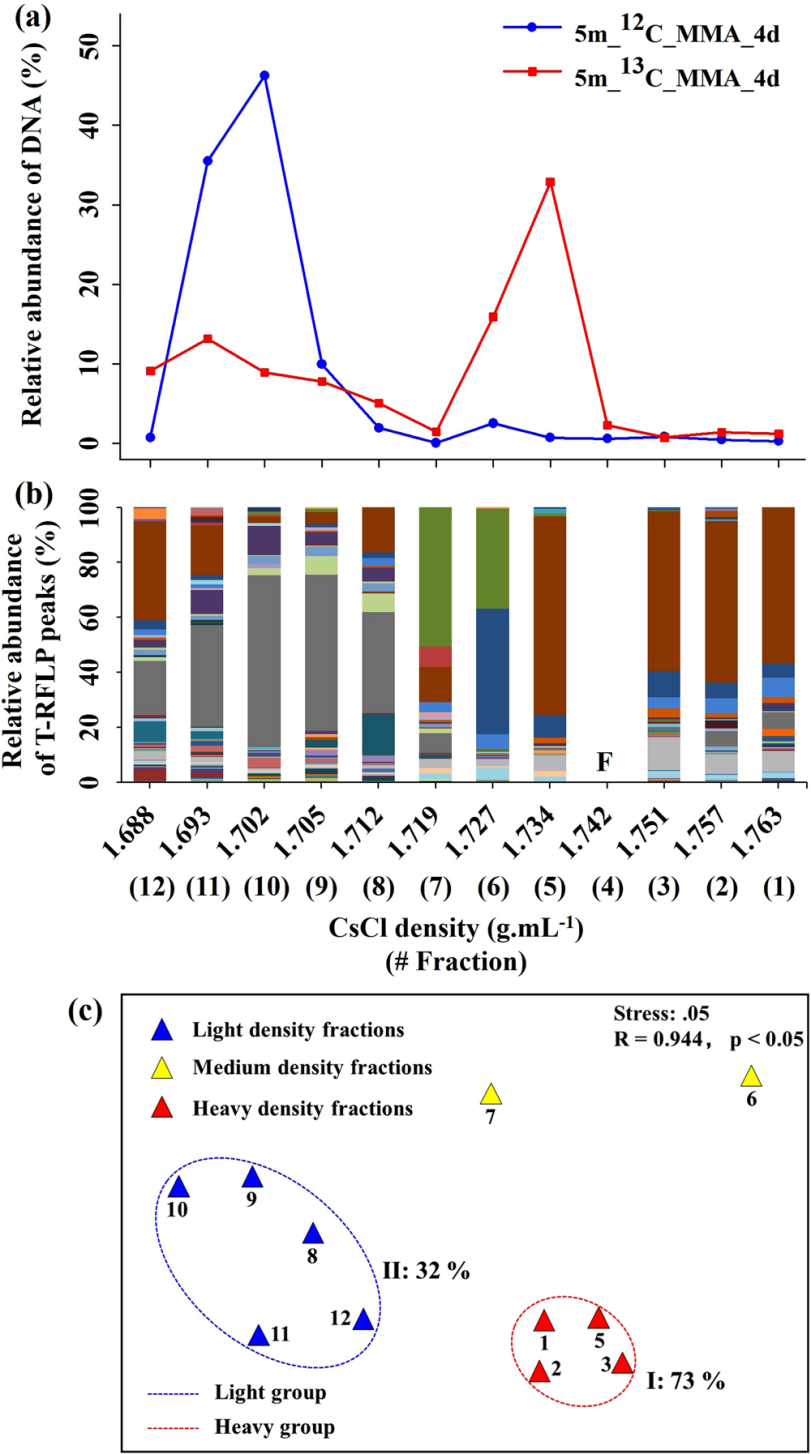
Bacterial community composition at 5 m water depth at site C3 based on T-RFLP analyses in density gradient fractions produced from ultracentrifugation of DNA after four days of incubation with supplemental ^13^C-labelled MOH. (**a**) Relative abundance of DNA in CsCl density gradients; (**b**) Relative abundance of T-RFLP peaks (colour bars) in each DNA fraction; (**c**) Nonmetric multidimensional scaling (NMDS) ordination based on the Bray-Curtis similarities between T-RFLP fingerprinting of bacterial communities in density gradient fractions. F = T-RFLP experiment failed.

Relative abundances of DNA in the CsCl density gradient fractions were analysed to locate ^13^C and ^12^C-DNA (Fig. 2a; Figs S1–S4). CsCl density gradually decreased from the 1^st^ to 12^th^ fraction, ranging from 1.687 to 1.770 g mL^−1^. Overall, DNA was distributed throughout all fractions, with one to two peak values in heavy and/or light density fractions. The DNA concentration peaks from incubations with supplemental ^13^C-labelled MOH at the estuary site P1 samples (5 m depth) were clearly distinguished from incubations with ^12^C-labelled MOH after one day. However, peaks were clearly distinguishable between ^13^C and ^12^C-DNA after four days of incubation of samples at the coastal site C3 (5 m depth), shelf site S2 (5 and 69 m depth), and 200 m of the central basin site B4 and two days at 5 m depth of site B4. For incubations with supplemental ^13^C and ^12^C-labelled MMA, ^13^C-DNA can be clearly distinguished after one day of incubation at 5 m depth of both sites C3 and B4 and after four days for sites S2 (5 and 69 m depth) and 200 m of site B4 (Figs S1–S4).

### Bacterial communities in density gradient fractions based on T-RFLP analyses

Bacterial community composition in the density gradient fractions, produced from ultracentrifugation of DNA retrieved from incubation with supplemental ^13^C-labelled substrates, was analysed with T-RFLP. The T-RFLP results (Fig. 2b) showed that there was a significant difference in T-RFs between heavy (H) and light (L) fractions for each sample. Figure 2 shows the sample from the ^13^C-MOH supplemented seawater incubation experiment at site C3 (5 m depth) as an example. Nonmetric multidimensional scaling (NMDS) ordination based on the Bray-Curtis similarities between T-RFLP fingerprinting of bacterial communities further revealed that community was separated into two major clusters: group I contained fractions 1–3 and 5 (heavy group, Bray-Curtis similarity = 73%), and group II contained fractions 8–12 (light group, 32%) (Fig. 2c). Meanwhile, fractions 5 and 10 were located at the ^13^C and ^12^C-DNA concentration peaks, respectively (Fig. 2a). Based on the analysis, fractions 5 and 10 obtained from the incubation with supplemental ^13^C-labelled substrate at site C3 (5 m depth) were selected as the representative H and L fraction, respectively, and used for further sequencing. By this means the representative H and L fraction of other samples were also selected. The fractions selected for each sample are shown in Table S2.

### Time-series analysis of substrate-incorporating bacterial communities

**Figure 3:**
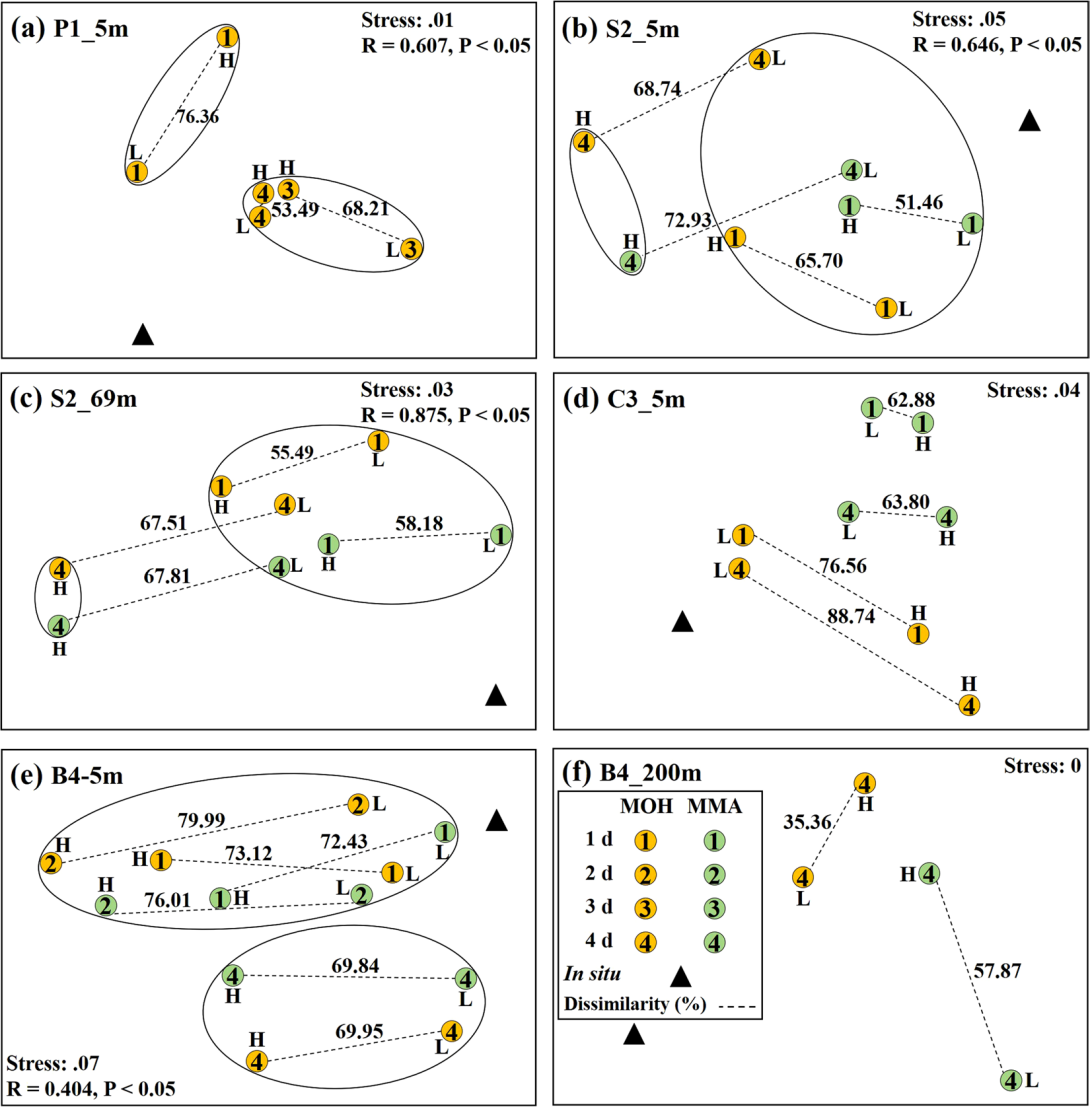
Nonmetric multidimensional scaling (NMDS) ordination based on Bray-Curtis dissimilarities between bacterial communities in the representative heavy (H) and light (L) fractions from all samples. Each circle represents an individual community in the NMDS charts. The number in the circle represents incubation time. The dashed line indicates the Bray-Curtis dissimilarity between two communities. The ellipse indicates a cluster. There was a statistically significant difference (ANOSIM test, P value  < 0.05) between two clusters. Incubation experiment of sample from (**a**) site P1 (5 m water depth); (**b**) site S2 (5 m water depth); (**c**) site S2 (69 m water depth); (**d**) site C3 (5 m water depth); (**e**) site B4 (5 m water depth); (**f**) site B4 (200 m water depth).

The representative H and L fractions from all samples were analysed with high-throughput sequencing to identify the active substrate-incorporating bacterial populations and populations that did not incorporate substrate. NMDS was built from the Bray-Curtis dissimilarities, which were calculated based on the relative abundance of OTUs, to discriminate bacterial community differences between the H and L fractions over the incubation time. For site P1 (Fig. 3a), the dissimilarity of bacterial communities at 5 m depth between H and L were 76.36%, 68.21%, and 53.49% after one, three, and four days of incubation, respectively. Moreover, bacterial community structure after one day of incubation was significantly different from three and four-day incubation periods (R = 0.607, P <0.05). Combining the community structure analysis and ^13^C and ^12^C-DNA concentration distributions in the density gradient fractions (Fig. S1), the sample at estuary site P1 after one day of incubation more accurately reflected the substrate-incorporating activity of the *in-situ* community. It also escaped the “bottle effect” and cross-feeding effect. In this context, the “bottle effect” alludes to an apparent reaction of bacteria to batchwise incubation in a confined environment (see Discussion)^39^. Cross-feeding is the phenomenon that one species lives off the products of another species, which would cause the detection of microorganisms that utilized a labelled intermediate produced by the primary incorporator of the substrate or dead biomass of primary incorporator in long periods of incubation^33^. Here, gradual homogenisation between H and L communities after three and four days of incubation could reflect this effect. In contrast, samples at shelf site S2 (both 5 and 69 m water depth) and coastal site C3 (5 m depth) (Fig. 3b‒d) showed dissimilarity in bacterial communities between H and L fractions after four days of incubation with both MOH and MMA higher than after one day. Statistically significant differences were observed between bacterial community structure in the H fractions after four days of incubation and the L fractions and after one-day incubation at site S2 (Fig. 3b,c; 5 m depth: R = 0.646, P <0.05; 69 m depth: R = 0.875, P <0.05). Combining this community structure analysis and ^13^C and ^12^C-DNA concentration distributions in the density gradient fractions (Figs S2 and S3), the samples at sites S2 and C3 after four days of incubation more appropriately reflected the active substrate-incorporating populations from the *in-situ* community. They also escaped the indistinguishable state between ^13^C and ^12^C-DNA that resulted from the short one-day incubation, but the influences of the “bottle effect” and the cross-feeding effect cannot be assessed. The dissimilarity of the bacterial community between H and L fractions from samples of the basin site B4 (5 m depth) after two-day incubation with both MOH and MMA was higher than one and four days (Fig. 3e), and meanwhile the bacterial community structure after four days of incubation was significantly different from after one and two days (R = 0.404, P <0.05). Taken together, dissimilarity of the bacterial community between H and L fractions and ^13^C- and ^12^C-DNA concentration distributions in the density gradient fractions (Fig. S4a‒d) indicated that the sample after two days of incubation more appropriately reflected the active substrate-incorporating populations from the *in-situ* community than one or four days of incubation. The sample escaped the indistinguishable state between ^13^C- and ^12^C-DNA that resulted from the short one-day incubation as well as the influences of the “bottle effect” and cross-feeding effect that resulted from the long four-day incubation. The sample at 200 m water depth of site B4 was only collected after four days of incubation (Fig. 3f). In almost all of the samples, the Bray-Curtis similarity of the bacterial community between the L fractions and their corresponding *in-situ* sample was higher than between the H fraction and *in-situ* sample (Table S3).

### Comparison among active substrate-incorporating bacterial communities

**Figure 4:**
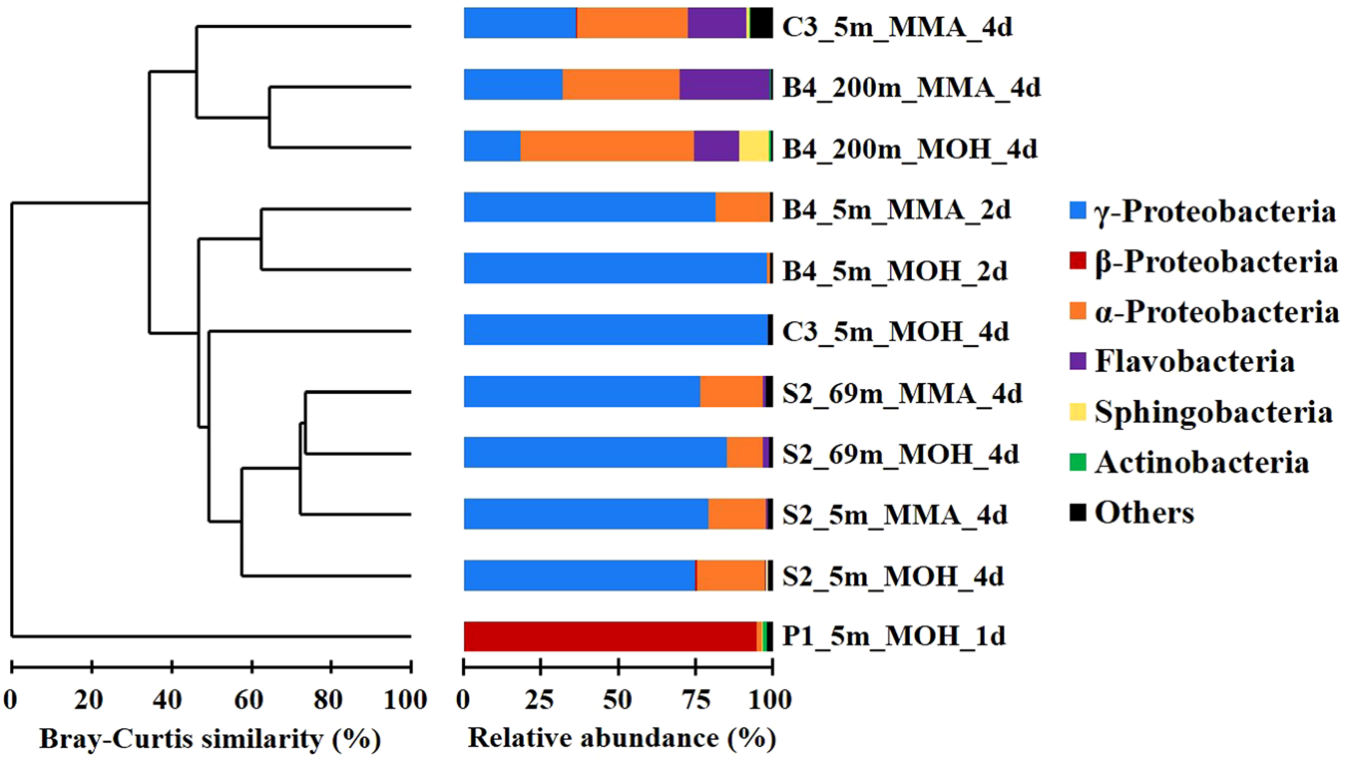
Dendrogram of the group average model based on Bray-Curtis similarities between the representative ^13^C-DNA communities. Colour bars indicate relative abundances of phylogenetic taxa in each library. MOH = methanol; MMA = monomethylamine; d = day.

H fractions of samples from appropriate incubation times were selected (Table S4) to analyse the phylogenetic composition of the active MOH and MMA-incorporating bacterial communities; the corresponding L fractions were also sequenced. Shannon diversity index values showed that ^13^C-DNA bacterial community from the incubations with MOH (0.93‒2.39, average = 1.61 ± 0.55) and MMA (1.38‒2.33, average = 1.75 ± 0.46) were significantly less diverse than the ^12^C-DNA and *in-situ* bacterial community (MOH: 1.86‒2.98, average = 2.57 ± 0.45; MMA: 1.8‒2.98, average = 2.51 ± 0.44; *in-situ*: 1.61‒2.75, average = 2.38 ± 0.45). This indicates that only a part of the bacterial population in the SCS is capable of incorporating supplemented C_1_ substrates^19^. Dendrogram analysis indicated that the active substrate-incorporating bacterial community structure (based on relative abundance of OTUs) at estuary site P1 is completely different (0% similarity) from other sites in the SCS (Fig. 4). *β*-Proteobacteria dominated the active MOH-incorporating populations at site P1 (94.7% of total reads), whereas the MOH and MMA-incorporating communities were dominated by *γ*-Proteobacteria (18.4%‒98.2%) and *α*-Proteobacteria (0.2%‒56.2%) in the SCS. At coastal site C3, the active MOH and MMA-incorporating bacterial community structures were different, with a 37.2% Bray-Curtis similarity, but they have a similarity of >56.1% at shelf site S2 and basin site B4. Notably at site B4, the active MOH and MMA-incorporating bacterial communities at 5 m water depth were distinct from 200 m water depth, with only a similarity of 37.2% (Fig. 4). The active communities at 200 m depth of site B4, as well as the MMA-incorporating community at 5 m depth of site C3, were co-dominated by *γ*-Proteobacteria (18.4%‒36.4%), *α*-Proteobacteria (36.1%‒56.2%), and *Flavobacteriia* (14.6%‒37.9%) (Fig. 4).

### Phylogenetic analysis of the active substrate-incorporating bacterial populations

**Figure 5:**
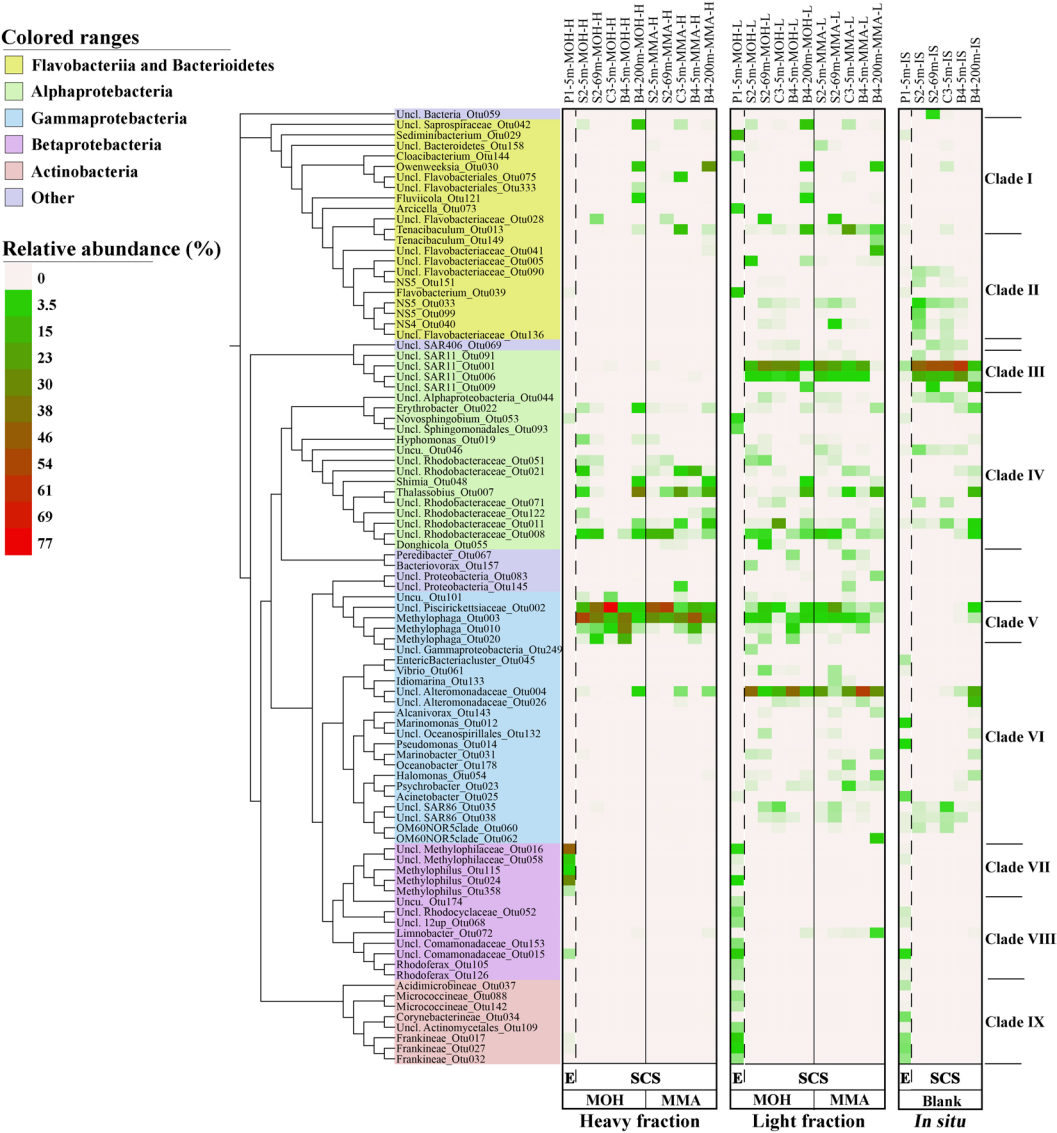
Phylo·genetic tree of OTU sequences with relative abundance>1% of total sequences in either the representative heavy (H) or light (L) fractions, or *in-situ* samples. Relative abundances of OTUs are shown as heat maps to the right of the phylogenetic tree. Figure was produced from the Interactive Tree Of Life (iTOL, http://itol.embl.de/). E, estuary; SCS, South China Sea.

A total of 91 representative sequences of OTUs with relative abundances >1% in either H or L fraction, or *in-situ* samples, were phylogenetically analysed and their relative abundances in each library were depicted with heat maps (Fig. 5). Overall, the active MOH and MMA-incorporating (^13^C-DNA) bacterial populations were mainly from the families *Methylophilaceae* (*β*-Proteobacteria) at estuary site P1 and *Piscirickettsiaceae* (*γ*-Proteobacteria) at the SCS sites. The ^12^C-DNA bacterial communities were more similar to *in-situ* communities, with major populations from *β*-Proteobacteria and *Actinobacteria* at site P1 and the SAR11 group of *α*-Proteobacteria at the SCS sites. In addition, members from *Alteromonadaceae* were abundant in the ^12^C-DNA communities.

**Figure 6:**
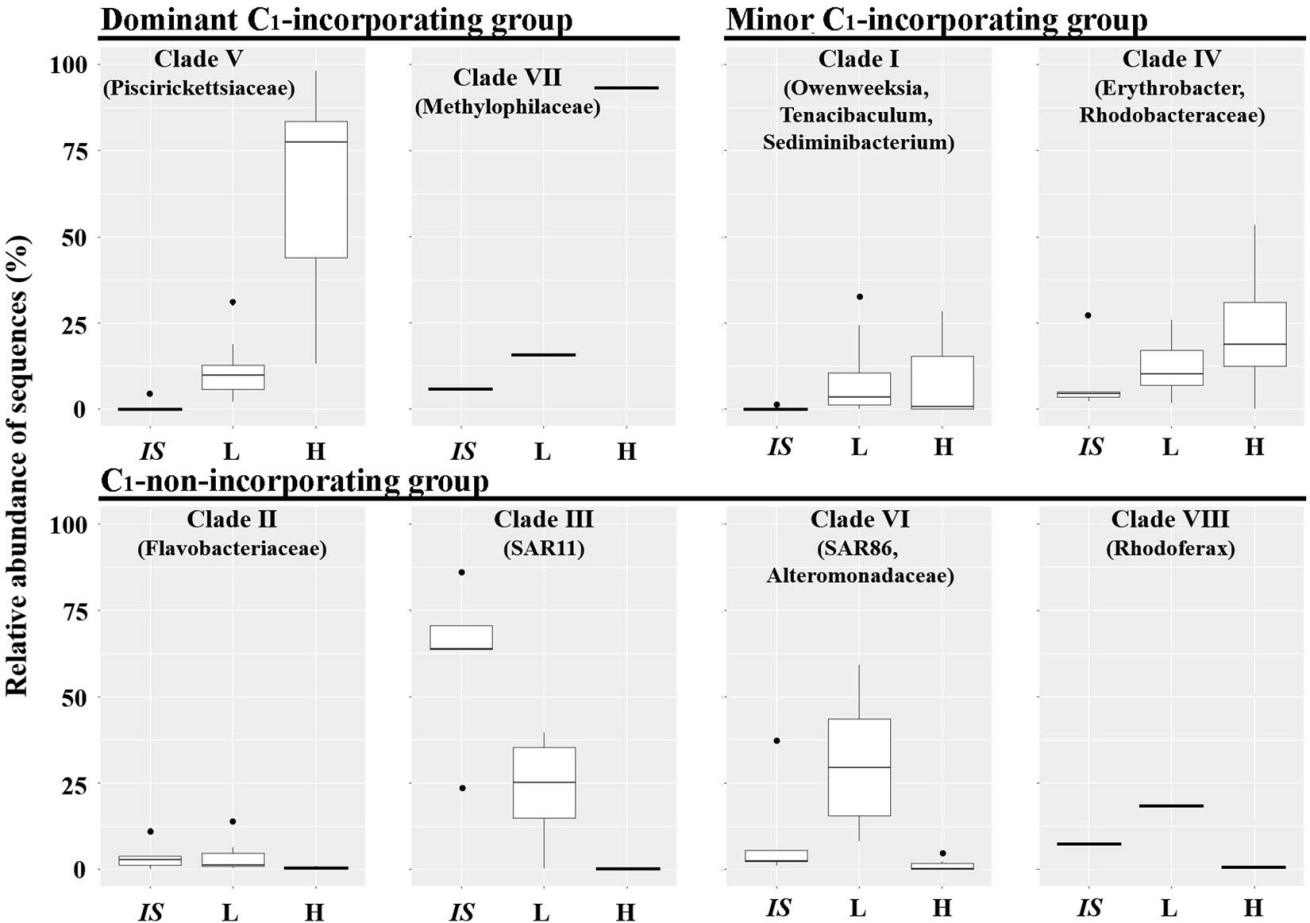
Models of typical bacterial ecological type response to the supplemented ^13^C-labelled substrates. Sequences were divided into nine clades (see Fig. 5). Boxplot indicates the relative abundance of OTU sequences in *in-situ* communities (IS), light (L), and heavy (H) fractions. The top and bottom boundaries of each box indicate 75^th^ and 25^th^ quartile values, respectively, and the black lines inside each box represent the 50^th^ quartile (median) values. The ends of the whiskers mark the lowest and highest values, excluding outliers (black circles).

Based on the framework of Nelson and Carlson^40^, we divided the OTUs into nine clades based on their relative abundance differences among H and L fractions, and *in-situ* samples (Fig. 5). Clade II (*Flavobacteriia*), Clade III (*α*-Proteobacteria), Clade VI (*γ*-Proteobacteria), Clade VIII (*β*-Proteobacteria) and Clade IX (*Actinobacteria*) did not incorporate ^13^C-labelled MOH and MMA, as the relative abundance of OTUs in these five clades were lowest in H fractions compared with L fractions and *in-situ* samples. Clade V (*γ*-Proteobacteria) was the dominant MOH and MMA-incorporating group in the SCS sites (S2, C3, and B4), whilst Clade VII (*β*-Proteobacteria) was the dominant MOH-incorporating group in the estuary site (P1). Clade I (*Flavobacteriia* and *Bacteroidetes*) and Clade IV (*α*-Proteobacteria) consist of diverse OTUs that incorporated ^13^C-labelled MOH and/or MMA; these OTUs were the minor MOH and MMA-incorporating groups with the similar relative abundance of OTUs in H and L fractions. Thus, the nine clades were classified as dominant, minor MOH and MMA-incorporating groups, and MOH and MMA-non-incorporating group. The representative phylogenetic taxa in the nine clades are shown in Fig. 6.

## Discussion

To clearly separate and obtain sufficient ^13^C-DNA under low substrate conditions, we supplemented the ^13^C-labelled substrates with a final concentration of 100 μM, which is higher than *in-situ* content. High substrate concentration has no influence on assessing whether a population incorporates it or not, although it might result in a “bottle effect” which could influence final cell concentrations^41^, viability/activity parameters^42^, cultivability^43^, and population composition^44^. Here, the difference between *in-situ* and ^12^C-DNA communities could reflect this effect. For instance, members from *Alteromonadaceae* were distinctly abundant in the ^12^C-DNA communities as compared to the *in-situ* communities. Members of *Alteromonadaceae* were the most frequently found lineages after incubation due to their capability of higher growth rate, faster response to nutrients, and better assimilation of carbon sources^45,46^.

The trade-offs between obtaining enough ^13^C-DNA and cross-feeding is a balancing act in SIP experiments. Thus, in this study, time-series incubations were performed to assess the best incubation time for accurate identification of substrate-incorporating populations. One day of incubation was enough to label sufficient DNA from samples of estuary site P1, four days were needed for SCS sites S2 and C3, and two days for site B4. This is broadly consistent with a previous study of samples from the English Channel that indicated MOH and MMA can be well incorporated after three or four days of incubation^6^. However, it is clear that the optimal incubation time varies in different environments, which is shorter in eutrophic estuarine waters than in oligotrophic open waters. Isotope ratio analysis also indicated that the substrate uptake rate in estuary site P1 samples were at least one order of magnitude higher than shelf site S2 after one day of incubation. These results suggest that C_1_ substrates could be more quickly assimilated by microbes in fresh water than in the shelf water of the SCS, and thus estuaries could be important MOH and MMA sinks. Overall, MOH and MMA had similar bio-availabilities in the SCS shelf, as indicated by time-series incorporation rates and active community composition. However, in the coastal and open ocean basin surface water, DNA distribution in CsCl density gradient fractions (Figs S3 and S4) indicated that the microbial response to MMA may be faster than to MOH despite similar active bacterial populations. A possible explanation is that MMA can act as a nitrogen source for marine microbes besides carbon source, particularly in the nutrient-limiting oligotrophic environments (Table S1). MOH is mainly used as an energy source (degraded to CO_2_), and only a small part of its uptake is attributed to cellular carbon source (incorporation into cell material)^5,47^. MMA can be used as both an energy and carbon source^48^. ^13^C-DNA indicates incorporation of the substrate, whilst the ^12^C-DNA community might include the groups utilising MOH or MMA only as a source of energy, such as SAR11^49^, as well as the groups that did not incorporate MOH or MMA.

There are three ecological types of bacterial taxa, characterised according to their response to supplemented MOH and MMA: rapid incorporation (I, dominant C_1_-incorporating group), slow incorporation (II, minor C_1_-incorporating group), and no incorporation (III, C_1_-non-incorporating group). Most of the taxa were ecological type III, which lack the ability to assimilate MOH and MMA, but might have the capacity of degrading them to CO_2_^50^. Most ecological type I taxa were fairly rare in the SCS sites, but quickly responded to supplemented MOH and MMA. Ecological type II had more diverse bacterial taxa, which could incorporate either MOH, MMA, or native organic matter as indicated by their relative abundance in L fraction and *in-situ* communities; however, it is possible these ^13^C-DNA taxa were derived from ^12^C-DNA due to cross-feeding.

Members of *Methylophilaceae* (*β*-Proteobacteria), belonging to ecological type I, are major MOH-incorporating populations in the estuary. They are widespread in a diverse range of fresh water habitats, such as mud flats, rivers, lakes, and ponds, and capable of assimilating MOH or methylamine^51,52^. In the SCS, MOH and MMA-incorporating populations included more diverse bacterial taxa belonging to *γ*-Proteobacteria (ecological type I), *α*-Proteobacteria (type II), *Flavobacteriia* (type II), and *Sphingobacteria* (type II), of which *Methylophaga* (*γ*-Proteobacteria, *Piscirickettsiaceae*) was the most dominant group. These are widespread in a diverse range of salt water habitats and are involved in C_1_ compounds assimilation^5,6,53^. Members of *Methylophaga* use the ribulose monophosphate pathway to assimilate C_1_ compounds^6^ and have been defined as strictly aerobic methylotrophs. The second most dominant group was *Thalassobius* (ecological type II), a newly defined genus^54^ of the *Rhodobacteraceae* that has not been tested the capability to assimilate MOH and MMA under natural or culture conditions to-date. Other identified ecological type II MOH and MMA-incorporating groups belong to *Shimia*, *Erythrobacter*, and *Tenacibaculum*, but these have not been reported to incorporate MOH and MMA to-date. Many ^13^C-DNA OTUs belonged to unclassified *Piscirickettsiaceae* (2.5% to 78.9% of total reads, ecological type I), *Rhodobacteraceae* (type II), *Flavobacteriaceae* (type II), and *Saprospiraceae* (type II), and had quite high relative abundances in some samples. These results indicate that there are more diverse MOH and MMA-incorporating bacterial taxa in marine environments than assumed previously^55^. To gain insight on the phylogeny and physiology of these methylotrophic bacteria, further investigations combining DNA-SIP with metagenomics or metatranscriptomics should be conducted^50,56^.

This study combined DNA-SIP time-series incubation with isotope ratio analyses to investigate the bio-availabilities of typical C_1_ compounds, MOH and MMA, and the microbes responsible for the assimilation of these C_1_ compounds in different water masses of the Pearl River estuary, coast, shelf, and central basin of the SCS. Our findings demonstrate a substantial difference in the rates of MOH and MMA incorporation and bacterial taxa, particularly between the estuary and SCS. The estuary microbial community can more quickly incorporate MOH than the SCS shelf community, and *Methylophilaceae* (*β*-Proteobacteria) are major microbial sinks of MOH. MOH and MMA generally had similar bio-availabilities in the SCS shelf, based on comparable incorporation rates and similar active bacterial populations. However, microbial response to MMA may be faster than to MOH in the coastal and basin surface water of the SCS despite similar active bacterial populations. *Piscirickettsiaceae* (*γ*-Proteobacteria) are major MOH and MMA-incorporating bacteria in the SCS. *Thalassobius* (*α*-Proteobacteria) were predominant at 200 m water depth in the central basin. Moreover, unexpectedly diverse MOH and MMA-incorporating bacterial groups were identified, which suggests a more important role in the transformation of C_1_ compounds in marine environments than hitherto assumed.

## Methods

### Sampling

Seawater was collected from 5 m water depth (about 100 L total) at sites P1 and C3, 5 and 69 m (near the bottom) water depth at site S2 (200 L collected from each depth), and 5 and 200 m water depth at site B4 (100 L from each depth) by a SeaBird CTD (SBE 9/11 plus) equipped with 12 L Niskin bottles during two cruises in July–October 2013. Two litres of seawater for *in-situ* community DNA extraction were collected on a 0.2 μm filter (47 mm diameter, Millipore Sterivex filters, EMD Millipore Corp., Merck KGaA, Germany) at each of the sites. The filters were flash-frozen in liquid nitrogen for 10 min and subsequently stored at −80 °C until analysis. Seawater (500 mL) for *in-situ* microbial ^13^C content measurements was collected from sites P1 and S2 on 0.3 μm glass fibre filters (25 mm diameter, Advantec, Toyo Roshi Kaisha, Japan), which were pre-combusted for 4 h at 525 °C^57^. All of the glass fibre filters were frozen at −20 °C until analysis.

### Biogeochemical parameters

Temperature, salinity, and depth data were obtained from the CTD system. Water samples for inorganic nutrients were filtered through 0.45 μm cellulose acetate membranes and then analyzed onboard. Ammonium was analyzed by the indophenol blue spectrophotometric method^58^. Nitrite, nitrate, and silicate concentrations were measured with a four-channel continuous flow Technicon AA3 Auto-Analyzer (Bran-Lube GmbH, Germany)^59^. The basic physical and chemical parameters were showed in Table S1.

### Field-based incubation experiments

Seawater collected for incubation was immediately filtered through a 3 μm polycarbonate filter (254 mm diameter, Pall Life Science, USA) to remove macrophytoplankton and grazers^60^. Twenty litres of filtered seawater were incubated in 20 L polycarbonate bottles, previously washed with a 10% HCl solution and filtered seawater. 99% ^13^C-labelled or >98% ^12^C-labelled MOH or MMA (Sigma, USA) was added into each bottle to form a final substrate concentration of 100 μM, and 0.1% marine ammonium mineral salts medium was supplemented to stimulate the microbes to incorporate the ^13^C-labelled substrates^36,61^. Bottles were sealed with polypropylene closures and thermoplastic elastomer gaskets to prevent substrate volatilisation, then incubated in *in-situ* seawater flow-through incubators. The microbial community (5 L total of the incubated seawater) was collected on 0.2 μm filters (Millipore Sterivex filters, EMD Millipore Corp., Merck KGaA, Germany) with a suction pressure of <0.03 MPa at 0 to 2 day intervals after incubation for sites P1, S2, C3, and the 5 m water depth sample at site B4; the 200 m water depth sample at site B4 was only collected after four days of incubation. Filters were flash-frozen in liquid nitrogen for 10 min and subsequently stored at −80 °C until DNA extraction in the laboratory. Incubated seawater (500 mL) for microbial ^13^C content measurements was filtered through a 0.3 μm glass fibre filter after one day and four days of incubation at sites P1 and S2. The glass fibre filters were frozen at −20 °C until laboratory analysis.

### DNA extraction, CsCl density gradient ultracentrifugation, and gradient fractionation

DNA was extracted using the phenol-chloroform-isoamyl alcohol method^51^. Purified DNA was checked with a NanoDrop device (ND2000, Thermo Fisher Scientific, Inc., Waltham, MA, USA), and fluorometrically quantified using a Qubit dsDNA Assay Kit (Invitrogen, Thermo Fisher Scientific, USA) and Qubit 2.0 Fluorometer (Invitrogen, Life Technologies, Singapore). Ultracentrifugation was performed according to previous protocols with minor modifications^62^. Briefly, about 3 μg of DNA from each sample was mixed with a gradient buffer containing 0.1 M Tris, 0.1 M KCl, and 1 mM EDTA, after which the mixture was added to a CsCl solution (1.89 g mL^−1^) to a final density of 1.725 g mL^−1^. The final solutions were poured into 5.1 mL ultracentrifuge tubes and centrifuged at 140,000 × g (~37,700 rpm) in a vertical rotor (VTi 65.2, Beckman Coulter, Inc., Brea, CA, USA) at 20 °C for 69 h under vacuum^63^. After centrifugation, the solution was divided into 12 equal fractions as soon as possible and the densities of all fractions were determined as described by Zhang *et al*.^34^. DNA in each fraction was retrieved by adding two volumes of PEG solution (30% polyethylene glycol 6000, w/v, 1.6 M NaCl, and 20–40 μg of glycogen), resuspended in 35 μL of TE (10 mM Tris-HCl, 1 mM EDTA, pH 8.0), and then fluorometrically quantified.

### T-RFLP analyses

Fractions were analysed by T-RFLP according to previous procedures^64^. Bacterial 16S rRNA genes were PCR-amplified from each DNA fraction with primers 27F-FAM (5′-AGAGTTTGATCMTGGCTCAG-3′, 5′ end-labelled with the dye carboxy fluorescein) and 927R (5′-ACCGCTTGTGCGGGCCC-3′). PCR products were purified with the Agarose Gel DNA Extraction kit (Tiangen Biotech Co., Ltd., China)^34^. Purified PCR products were digested with FastDigest MspI and RsaІ (Thermo Fisher Scientific, Inc.) at 55 °C for 4‒12 h. Digested products were purified by precipitation with ethyl alcohol and then resuspended in 20 μL of sterile deionised water. Purified products were then mixed with 0.5 μL of an internal standard and detected on an Applied Biosystems Automated 3730 DNA analyser (Applied Biosystems, USA). T-RFLP data were analysed with MegaBACE software (Amersham Biosciences Corp., USA). The fragment profiles of the 12 density gradient fractions from each incubation experiment, as well as community similarity between fractions, were analysed using Primer 5 software.

### High-throughput sequencing and statistical analysis

Two fractions, corresponding to the ^13^C (heavy) and ^12^C (light), respectively, present in DNA from each sample were selected for high-throughput sequencing. The V3–V4 hypervariable region of bacterial 16S rRNA genes was amplified with the universal primers 341F (CCTACGGGNGGCWGCAG) and 805R (GACTACHVGGGTATCTAATCC)^65^. Sequencing was carried out using an Illumina MiSeq platform at the Chinese National Human Genome Center (Shanghai, China).

Quality controlled sequences were analysed with the standard operating procedure using mothur software (www.mothur.org/wiki/MiSeq_SOP)^66^. Briefly, the sequences were simplified to unique sequences with the *unique.seqs* command, and then aligned to the SILVA bacterial database using the *align.seqs* command. Then, *screen.seqs* and *filter.seqs* commands were applied to remove the sequences that had lengths outside the desired range and columns with only gaps. The sequences within 2 bp of difference to a more abundant sequence were merged with the *pre.cluster* command to reduce sequencing error. Chimeras were identified and removed using the *chimera.uchime* and *remove.seqs* commands, respectively. Finally, *classify.ses* and *remove.lineage* commands were run to remove reads that were classified as “Cyanobacteria_Chloroplast”, “Mitochondria” or could not be classified at the kingdom level. The confidence cut-off was set to 80%. Sequences were further clustered into Operational Taxonomic Units (OTUs) with a cut-off value of 0.03^67^. The taxonomy for each of the OTUs was detected by *classify.otu*. For further normalisation, sequences in all samples were rarefied and subsampled to an equal number using the *sub.sample* command. Bray-Curtis similarities were calculated based on OTU relative abundance matrices and the community similarity between fractions was analysed by nonmetric multidimensional scaling (NMDS) and dendrograms using Primer 5. One-way analysis of similarity (ANOSIM) with 999 permutations was performed to test the null hypothesis of no significant difference between clusters in the NMDS charts.

### Phylogenetic analysis

Representative sequences of each OTU were selected out through the *bin.seqs* and *get.oturep* commands for further phylogenetic analysis. Sequences were aligned and compiled using the MEGA7 program and the maximum likelihood phylogenetic tree was constructed.

### Microbial substrate assimilation

The amount of organic carbon retained from each glass fibre filter was measured according to the following methods^68,69^. Briefly, glass fibre filter samples were freeze-dried for at least 24 h, and then steamed in concentrated HCl for 48 h. Samples were placed in 5 × 9 mm tin cups and measured for particulate organic carbon (POC) in 1 L of incubated seawater (m_POC_). Carbon isotopic compositions (*δ*^13^C) were measured with an elemental analyser coupled to a stable isotope ratio mass spectrometer (Flash EA 1112 HT-Delta V Advantages, Thermo). Two reference materials (Acetanilide#1: *δ*^13^C = −29.53‰; Urea#2: *δ*^13^C = −8.02‰) were used to calibrate the δ^13^C_POC_ of the samples^70^. The analytical precision for δ^13^C_POC_ was better than ±0.2‰. The mass of ^13^C in microbial biomass was calculated using the equations:

1 $${R}_{sample}=(\frac{{\rm{\delta }}{}_{\,}{}^{13}{\rm{C}}_{POC}}{1000}+1)\times {R}_{ref}$$

2 $$m({}_{\,}{}^{13}C)=\frac{13\times {m}_{POC}\times {R}_{sample}}{13\times {R}_{sample}+12}$$

where *m*(^13^*C*) is the mass of microbially assimilated ^13^C in 1 L of incubated seawater, *R*_*sample*_ is the atomic percent of ^13^C in the total organic carbon of the incubated sample, *R*_*ref*_ is the atomic percent of ^13^C in the international reference material Vienna Peedee Belemnite (VPDB), and *R*_*ref*_ = 0.0112372^71^.

In this study, there were two sources of ^13^C in the microorganisms, from the natural environment and supplemented by the ^13^C-substrate. The microbial assimilation rate of ^13^C from the supplementation into cells was calculated using the equation:

3 $$Assimilation\,rate\,({}_{\,}{}^{13}C)=\frac{{m}_{sample}\,({}_{\,}{}^{13}C)\,-\,{m}_{blank}\,({}_{\,}{}^{13}C)}{t}$$

where *m*_*sample*_ (^13^*C*) and *m*_*blank*_ (^13^*C*) is the microbially assimilated ^13^C mass in 1 L of incubated seawater from the ^13^C and ^12^C-substrate supplemented incubations, respectively, and *t* is the incubation time. The percentage of ^13^C in the POC pool was calculated using the equation:

4 $$Percentage\,of\,{}_{\,}{}^{13}C\,in\,POC\,pool=\frac{{m}_{sample}\,({}_{\,}{}^{13}C)\,-\,{m}_{blank}\,({}_{\,}{}^{13}C)}{{m}_{POC}}$$

where *m*_*POC*_ is the total mass of POC.

## Electronic supplementary material

Supplementary information

## Data Availability

The sequencing data were deposited in NCBI Sequence Read Archive (SRA) with Accession Number SRP142566. The representative sequences used in the phylogenetic tree are under GenBank Accession Numbers MH121322 to MH121412.
