# Supplementary material for: Differential incorporation of one-carbon substrates among microbial populations identified by stable isotope probing from the estuary to South China Sea
Source: Sci Rep. 2018 Oct 18;8:15378. doi: 10.1038/s41598-018-33497-6 (PMC6194082; doi:10.1038/s41598-018-33497-6)
Supplement: Supplementary file 1 — Supplementary information [file 41598_2018_33497_MOESM1_ESM.docx]

**Supplementary Information**

**Differential incorporation of one-carbon substrates among microbial populations identified by stable isotope probing from the estuary to South China Sea**

Wenchao Deng, Lulu Peng, Nianzhi Jiao, Yao Zhang^*^

State Key Laboratory of Marine Environmental Sciences and College of Ocean and Earth Sciences, Xiamen University, Xiamen 361101, China

^*^Correspondence: Y.Z. ([yaozhang@xmu.edu.cn](mailto:yaozhang@xmu.edu.cn))

**Table S1 Basic water mass parameters of sampling stations.**

| Station | Depth | Temperature | Salinity | NO_2_^-^ + NO_3_^-^ | NH_4_^+^ | SiO_3_^2-^ |
| --- | --- | --- | --- | --- | --- | --- |
|  |  | ℃ |  | µmol L^-1^ | nmol L^-1^ | µmol L^-1^ |
| P1 | 5m | 29.10 | 0.14 | 148.03 | 34.42 | 158.41 |
| S2 | 5m | 29.87 | 30.93 | 1.41 | 66.26 | 4.27 |
|  | 69m | 27.14 | 33.50 | 9.33 | BLQ | 12.96 |
| C3 | 5m | 27.19 | 33.37 | BLQ | BLQ | 2.43 |
| B4 | 5m | 28.69 | 32.90 | BLQ | 7.08 | 2.53 |
|  | 200m | 14.53 | 34.50 | 17.77 | 34.80 | 22.78 |

BLQ represent Below the Limit of Quantitation.

**Table S2 Heavy (^13^C-DNA) and light (^12^C-DNA) fractions selected from each sample for high-throughput sequencing.**

| Station | Sample | Heavy | Light |
| --- | --- | --- | --- |
| P1 | 5m_MOH_1d | 5 | 9 |
|  | 5m_MOH_3d | 6 | 10 |
|  | 5m_MOH_4d | 6 | 8 |
| S2 | 5m_MOH_1d | 4 | 8 |
|  | 5m_MOH_4d | 4 | 8 |
|  | 5m_MMA_1d | 4 | 8 |
|  | 5m_MMA_4d | 4 | 9 |
|  | 69m_MOH_1d | 4 | 8 |
|  | 69m_MOH_4d | 5 | 9 |
|  | 69m_MMA_1d | 4 | 10 |
|  | 69m_MMA_4d | 4 | 9 |
| C3 | 5m_MOH_1d | 4 | 9 |
|  | 5m_MOH_4d | 5 | 10 |
|  | 5m_MMA_1d | 6 | 9 |
|  | 5m_MMA_4d | 5 | 9 |
| B4 | 5m_MOH_1d | 3 | 9 |
|  | 5m_MOH_2d | 5 | 10 |
|  | 5m_MOH_4d | 4 | 8 |
|  | 5m_MMA_1d | 5 | 10 |
|  | 5m_MMA_2d | 5 | 10 |
|  | 5m_MMA_4d | 4 | 9 |
|  | 200m_MOH_4d | 2 | 11 |
|  | 200m_MMA_4d | 2 | 8 |

**Table S3 Bray-Curtis similarities of bacterial communities between *in situ* samples and the heavy (^13^C-DNA) or light (^12^C-DNA) fraction.**

| Site and depth | Sample | Heavy | Light |
| --- | --- | --- | --- |
| P1_5m | MOH_1d | 8.2 | 23.83 |
|  | MOH_3d | 17.74 | 10.56 |
|  | MOH_4d | 17.38 | 19.47 |
| S2_5m | MOH_1d | 16.41 | 27.66 |
|  | MOH_4d | 2.25 | 14.88 |
|  | MMA_1d | 35.06 | 36.48 |
|  | MMA_4d | 2.64 | 34.71 |
| S2_69m | MOH_1d | 24.35 | 31.83 |
|  | MOH_4d | 5.4 | 30.82 |
|  | MMA_1d | 36.2 | 39.28 |
|  | MMA_4d | 4.59 | 31.03 |
| C3_5m | MOH_1d | 15.65 | 33.35 |
|  | MOH_4d | 3.04 | 38.85 |
|  | MMA_1d | 10.5 | 10.35 |
|  | MMA_4d | 8.46 | 18.94 |
| B4_5m | MOH_1d | 15.95 | 35.07 |
|  | MOH_2d | 4.26 | 37.61 |
|  | MOH_4d | 18.17 | 26.11 |
|  | MMA_1d | 23.11 | 47.44 |
|  | MMA_2d | 10.15 | 33.64 |
|  | MMA_4d | 26.02 | 36.09 |
| B4_200m | MOH_4d | 35.73 | 44.54 |
|  | MMA_4d | 33.41 | 35.23 |

**Table S4 Optimal incubation time for each sample.**

| Station | Depth (m) | Appropriate incubation time (days) | |
| --- | --- | --- | --- |
|  |  | MOH | MMA |
| P1 | 5 | 1 | N |
| S2 | 5 | 4 | 4 |
|  | 69 | 4 | 4 |
| C3 | 5 | 4 | 4 |
| B4 | 5 | 2 | 2 |
|  | 200^a^ | 4 | 4 |

^a^Sample was only collected after four-day incubation

N, No experiment was carried out.

**Supplementary Figure S1. Relative abundances of DNA in CsCl density gradient fractions for each incubation experiment sample with supplemental ^13^C labelled methanol (MOH) from 5 m water depth of site P1.** (a) After one day of incubation; (b) After 3 days incubation; (c) After four-days incubation. CsCl density gradually increased from the 12^th^ to 1^st^ fraction.

**Supplementary Figure S2. Relative abundance of DNA in CsCl density gradient fractions for each incubation experiment sample from site S2.** Samples at 5 m water depth (a) after one day of incubation with supplemental ^13^C-labelled methanol (MOH), (b) after four days with supplemental ^13^C-MOH, (c) after one day with supplemental ^13^C-labelled monomethylamine (MMA), and (d) after four days with supplemental ^13^C-MMA. Samples at 69 m water depth (e) after one day with supplemental ^13^C-MOH and (f) after four days with supplemental ^13^C-MOH, (g) after one day with supplemental ^13^C-MMA and (h) after four days with supplemental ^13^C-MMA. CsCl density gradually increased from the 12^th^ to 1^st^ fraction.

**Supplementary Figure S3 Relative abundance of DNA in CsCl density gradient fractions for each incubation experiment sample from 5 m water depth of site C3.** (a) After one day of incubation with supplemental ^13^C-labelled methanol (MOH); (b) After four days with supplemental ^13^C-MOH; (c) After one day with supplemental ^13^C-labelled monomethylamine (MMA); (d) After four days with supplemental ^13^C-MMA. CsCl density gradually increased from the 12^th^ to 1^st^ fraction.

**Supplementary Figure S4. Relative abundance of DNA in CsCl density gradient fractions for each incubation experiment sample from site B4.** Samples from 5 m water depth (a) after one day of incubation with supplemental ^13^C-labelled methanol (MOH), (b) after 2 days with supplemental ^13^C-MOH, (c) after one day with supplemental ^13^C-labelled monomethylamine (MMA), and (d) after 2 days with supplemental ^13^C-MMA. Samples from 200 m water depth after four days of incubation (e) with supplemental ^13^C-MOH and (f) with supplemental ^13^C-MMA. CsCl density gradually increased from the 12^th^ to 1^st^ fraction.
